# A new mechanistic model of weather-dependent Septoria tritici blotch disease risk

**DOI:** 10.1098/rstb.2018.0266

**Published:** 2019-05-06

**Authors:** Thomas M. Chaloner, Helen N. Fones, Varun Varma, Daniel P. Bebber, Sarah J. Gurr

**Affiliations:** Department of Biosciences, University of Exeter, Stocker Road, Exeter EX4 4QD, UK

**Keywords:** *Zymoseptoria tritici*, Septoria tritici blotch, weather, mechanistic, modelling, infection risk

## Abstract

We present a new mechanistic model for predicting Septoria tritici blotch (STB) disease, parameterized with experimentally derived data for temperature- and wetness-dependent germination, growth and death of the causal agent, *Zymoseptoria tritici*. The output of this model (A) was compared with observed disease data for UK wheat over the period 2002–2016. In addition, we compared the output of a second model (B), in which experimentally derived parameters were replaced by a modified version of a published *Z. tritici* thermal performance equation, with the same observed disease data. Neither model predicted observed annual disease, but model A was able to differentiate UK regions with differing average disease risks over the entire period. The greatest limitations of both models are: broad spatial resolution of the climate data, and lack of host parameters. Model B is further limited by its lack of explicitly defined pathogen death, leading to a cumulative overestimation of disease over the course of the growing season. Comparison of models A and B demonstrates the importance of accounting for the temperature-dependency of pathogen processes important in the initiation and progression of disease. However, effective modelling of STB will probably require similar experimentally derived parameters for host and environmental factors, completing the disease triangle.

This article is part of the theme issue ‘Modelling infectious disease outbreaks in humans, animals and plants: approaches and important themes’. This issue is linked with the subsequent theme issue ‘Modelling infectious disease outbreaks in humans, animals and plants: epidemic forecasting and control’.

## 1. Introduction

Crop plants are threatened by a plethora of pathogens [1]. Despite fungicide application and deployment of disease-resistant cultivars, such pathogens destroy around one-quarter of food production worldwide [2]. Wheat is the most important global cereal crop [3]; in the UK alone, over 12 million tonnes of wheat were harvested in 2013–2014 [4]. However, UK wheat yields are reduced up to 10% by the fungal pathogen *Zymoseptoria tritici,* the causal agent of Septoria tritici blotch (STB) [4], despite a spend of around £145 million (2014) on fungicides for its control [4,5].

Wind-dispersed ascospores are the primary inoculum of *Z. tritici* [6]. In moist conditions, spores germinate and hyphae grow randomly across the leaf, before entering via stomata [7–9]. Penetration and apoplast colonization precede an exponential increase in fungal biomass via necrotrophic growth *in planta* [7]. Pycnidia (asexual fruiting-bodies) then form and exude pycnidiospores, which are disseminated by rain-splash [9,10]. This enables polycyclic infection throughout the growing season [11]. Successful completion of the *Z. tritici* life cycle is dependent on complex, interacting factors, including temperature, moisture and light [12–18].

Both correlative and mechanistic attempts have been made to model STB disease risk and to develop disease forecasting tools based on weather data [19,20]. The correlative approach involves searching for relationships between disease severity and weather data [21–23]. Using this approach, ‘very severe’ or ‘severe’ STB epidemics were found to follow, respectively, 40 or 34 days of rain and warmth in late spring. This is in line with the observation that rainfall is crucial for temperature-dependent polycyclic disease progression [22]. A predictive model, based on accumulated rainfall and minimum temperatures during key stages of wheat growth, was moderately successful at predicting the binary presence/absence of a ‘damaging epidemic’: 63% of positive predictions and 73% of negative predictions matched epidemic observations overall. However, these numbers were subject to change depending on the STB resistance of the wheat cultivar, with negative predictions no better than random (50%) for susceptible cultivars [23]. This demonstrates the main weakness of correlative models: they do not account for the biology of the pathogen or host. By contrast, mechanistic models include host and/or pathogen parameters, such as temperature responses [24–27]. Two climate-focused, mechanistic models exist for STB [24,25]: both rely on pathogen parameters from unpublished data and relatively simple descriptions of pathogen development. For example, Audsley *et al*. [24] modelled the overall rate of lesion development as a function of temperature. Ideally, however, mechanistic models should account for complexities in pathogen temperature responses, such as variation in pathogen temperature responses during pathogen development or disease progression. The recent mechanistic model for *Hemileia vastatrix* (coffee rust) [19], in which germination and appressorium formation occur with different temperature optima [28], exemplifies this, but such detailed mechanistic models are yet to be created for *Z. tritici*.

**Figure 1.**
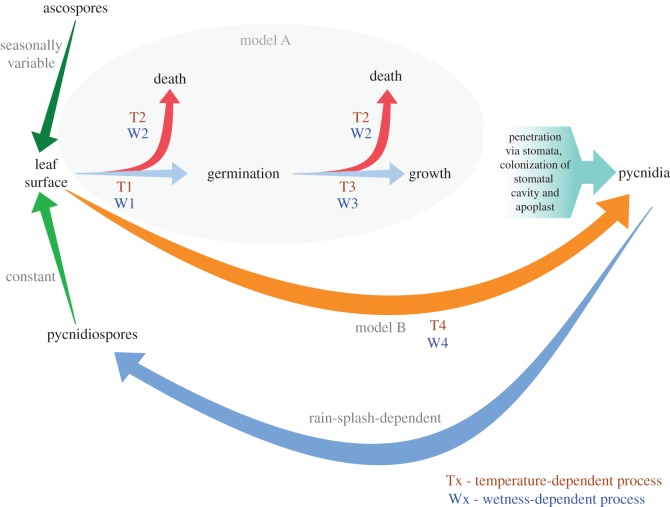
Schematic of models A and B. Models A0, A1 and A2 are described by experimentally derived parameters for the temperature- and leaf wetness-driven transition probabilities of spore germination (T1, W1), spore death (T2, W2), and hyphal growth (T3, W3). Model B1 is described by the temperature- and leaf wetness-driven transition probabilities between spores landing on the leaf surface and pycnidiation (T4, W4); model B2 is only described by T4 [29]. (Online version in colour.)

Here, we develop a new mechanistic model for STB (model A). We present experimentally derived parameters for the temperature- and leaf wetness-driven transition probabilities of (1) germination, (2) hyphal growth and (3) spore death (figure 1, T1–T3 and W1–W3; electronic supplementary material, figure S1). This model is thus based upon discrete probabilistic events during disease development. We modelled infection risk by running model A with a high-resolution climate dataset (1990–2016) [30]. In order to determine what value the experimentally derived parameters used in model A provided, we compared the performance of model A with a second version of this model (B) in which the experimentally parameterized, discrete, probabilistic events of disease development in model A were replaced by a thermal performance equation for *Z. tritici* developed by Bernard *et al.* [29]. This thermal performance equation defines a relationship between temperature and the latent period (LP) of STB disease [29]. Therefore, in model B, disease severity is determined by a direct, temperature-driven probability that spores landing on the leaf lead to pycnidiation (figure 1, T4; electronic supplementary material, figure S2). We compared the output of models A and B with observed STB data.

## 2. Material and methods

For details of *Z. tritici* strains and experimental conditions, preparation of leaf wax slides, calculation of *Z. tritici* temperature-dependent *in vitro* growth and germination rates, and *Z. tritici* temperature- and wetness-dependent thermal death rates (see electronic supplementary material; Material and methods).

### (a) Infection risk model parameterization

To parameterize model A, cardinal (minimum (*T*_min_), maximum (*T*_max_) and optimal (*T*_opt_)) temperatures (CT) for germination and growth were estimated by brute force fitting (an exploration of parameter space in which 10 million possible parameter combinations were considered and those that produced the fit with the smallest residual sums of squares selected) of the experimental data to a four parameter beta function (*T*_min_, *T*_max_, *T*_opt_ and scaling factor), after Yan & Hunt [31]. For growth, CT estimates were also shifted according to the relationship described in Boixel *et al.* [32], between the optimal temperature for growth *in vitro* and that for disease progression in leaves. This shift was used because, while we measured growth *in vitro*, it is the process of leaf infection that is modelled. Boixel *et al.* [32] also showed that the optimal *in vitro* growth temperature for IPO323 was 5.5°C higher than that for recently collected French field isolates of *Z. tritici.* To account for this, we used a third set of CTs for growth, those reported for field isolates—again, shifted for the difference between *in vitro* growth and leaf infection. CT parameter restrictions, estimates and shifted estimates are given in table 1. Hereafter, ‘model A0’ refers to the model using the growth CT parameters shifted to give optimal infection temperatures. Unshifted CT parameters are used in model A1, and shifted field isolate parameters in model A2. The results of models A1 and A2 are presented as supplementary results (electronic supplementary material, figures S3 and S4). CT parameters are assumed not to differ between ascospores and pycnidiospores in models A0, A1 and A2.

**Table 1.** Model restrictions and estimates for temperature parameters describing germination and growth for models A0, A1 and A2. Parameter estimation for germination (models A0, A1 and A2) and growth (A1) were restricted during brute force fitting of 4-parameter beta curves to experimental data. For A1/A2 model outputs (shaded columns), see electronic supplementary material, figures S3 and S4.

	germination		growth (stomatal penetration)			
	models A0, A1 and A2		model A1		model A0	model A2
parameter	restriction	estimate	restriction	estimate	estimate^a^	estimate^b^
minimum temperature (*T*_min_)	−5 to 10°C	9.92°C	−5 to 15°C	−4.52°C	−10.35°C	0.74°C
optimum temperature (*T*_opt_)	12 to (*T*_max_−0.01°C)	19.10°C	18 to (*T*_max_−0.01°C)	23.00°C	17.17°C	16.14°C
maximum temperature (*T*_max_)	25 to 35°C	32.20°C	25 to 35°C	25.60°C	19.77°C	29.34°C
scaling factor	0–0.5	0.09	0–10	8.57	8.57	8.57

^a^Parameter estimates calculated as model A1 parameter estimates, minus 5.83°C. This is to reflect differences between *Z. tritici in vitro* growth and area under disease progress curve (AUDPC), see [32].

^b^Parameter estimates are calculated as the mean of 18 *Z. tritici* isolates, taken directly from [32], minus 4.156°C. This is to reflect differences between *Z. tritici in vitro* growth and AUDPC, see [32].

**Figure 2.**
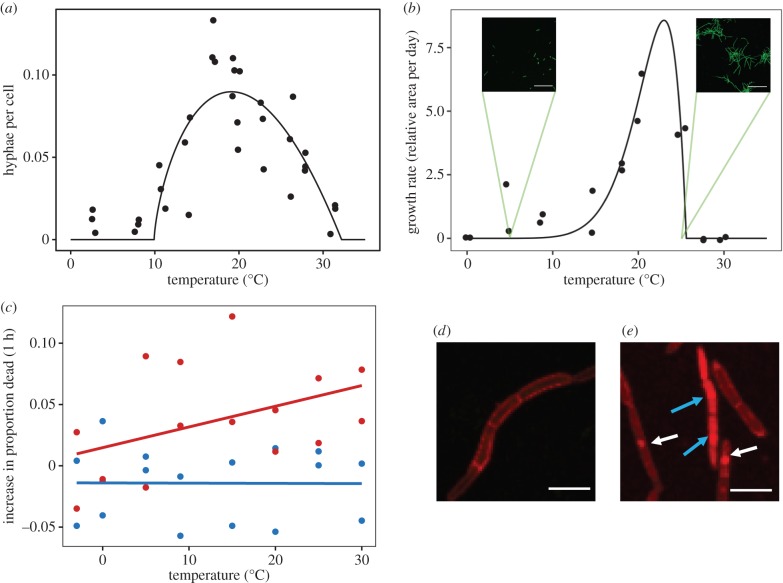
Model A parameterization. Temperature response functions for (*a*) germination and (*b*) hyphal growth. *T*_min_, *T*_opt_ and *T*_max_ of temperature response functions were calculated by brute force fitting of the observed data to a beta function (10 million brute force iterations), such that residual sums of squares (RSS) were minimized. RSS for (*a*) germination and (*b*) growth were calculated as 0.0141 and 9.24, respectively. Insets show representative images of *Z. tritici* after 15 days growth at 5°C (left) or 25°C (right). (*c*) Increase in proportion of cells dying during 1 h under wet (blue) and dry (red) conditions. Lines show linear regression analysis. Slope of blue line = −0.00178, s.e. = 0.069, *t* = −0.026, d.f. = 14, *p* = 0.980; slope of red line = 0.169, s.e. = 0.092, *t* = 1.830, d.f. = 14, *p* = 0.0881. (*d*,*e*) Representative images of blastospores stained with propidium iodide (*d*) immediately after inoculation (wet) or (*e*) after 4 h drying at 18°C. Fluorescence of propidium iodide within the entire cell (blue arrows), or nuclear fluorescence (white arrows) indicated cell death. Scale bars in (*b*) represent 100 µm; (*d* and *e*) represent 10 µm.

Cumulative germination and growth on the leaf were modelled using a cumulative Weibull distribution [19]. Growth is therefore considered as an event that has, as its end point, stomatal penetration. The Weibull parameters used for germination and growth were *α* = 58.5, *γ* = 1.3, and *α* = 189, *γ* = 2.2. Weibull parameters were chosen based on iterative fitting to available data, with the *α* and *γ* that resulted in the smallest residual sum of squares selected. Spore germination data were extracted from Fones *et al*. [7]. Only the first 5 days post-inoculation (dpi) was used, as this captured the majority of spore germination. The data were rescaled between 0 and 1, where the greatest percentage germination at day 5 was set to 1; see electronic supplementary material, figure S5. Growth (stomatal penetration) data for the first 0–10 dpi period was extracted from Fones *et al*. [7], with the additional assumptions that (1) the first 48 h of data reflect spore germination, and so were excluded from the dataset, and (2) at 8 dpi (10 dpi raw data) the mean number of germinated individuals penetrating stomata was 66% of the total. The data were then rescaled between 0 and 1; see the electronic supplementary material, figure S5. Such assumptions gave 90% penetration saturation at 11.5 days and 99% penetration saturation at 15.8 days under optimal conditions. Germination and penetration rates were reduced at sub-optimal temperatures according to their beta functions (figure 2*a*–c).

**Figure 3.**
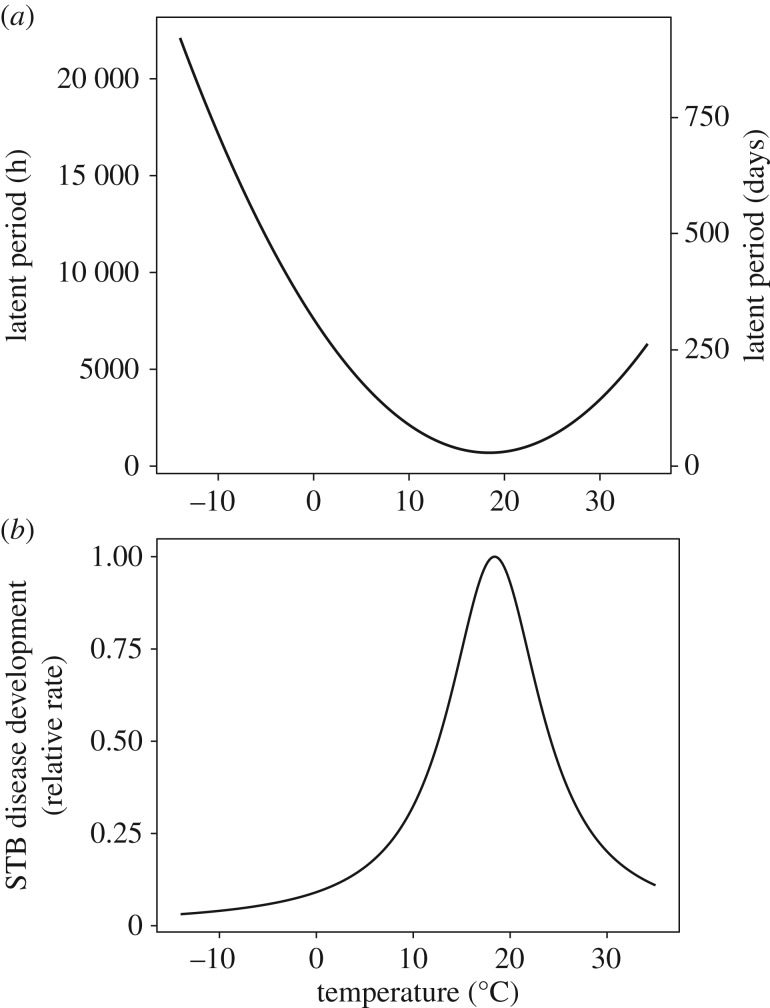
Model B parameterization. (*a*) Average effect of temperature on the latent period (time elapsed from inoculation until 37% of maximum by area sporulation) of three French isolates of *Z. tritici*. Calculated as the inverse of equation (2.1), taken from Bernard *et al*. [29]. (*b*) Temperature-dependent relative rate of STB disease development, calculated from equation (2.1) and rescaled (0–1), allowing temperature-dependent relative rates of STB disease development to be calculated.

In addition, model A included parameters for temperature-dependent spore death under both wet and dry conditions. The per-hour increase in proportional cell death was not influenced by temperature under wet conditions (linear regression, slope = −0.00178, s.e. = 0.069, *t* = −0.026, d.f. = 14; figure 2*c*, blue) but was positively related to temperature under dry conditions (linear regression, slope = 0.169, s.e. = 0.092, *t* = 1.830, d.f. = 14; figure 2*c*, red). Hence, we parameterize model A with zero spore death under wet conditions (irrespective of temperature) and predicted temperature-dependent spore death under dry conditions linearly, with a slope of 0.169. In addition, we found that the proportion of dead cells was greater by 0.3612 (s.d. = 0.028, d.f. = 2) in freshly prepared dry slides than in freshly prepared wet slides (a difference in wet and dry ‘baseline’ values; see electronic supplementary material; Material and methods), a difference we attribute to the stress of drying *per se.* The model therefore includes this additional spore death at the beginning of each dry spell in the climate data. We note that the linear fit for the proportion of dead cells under dry conditions generates negative predictions at very low temperatures, outside of the experimental data space. These temperatures (less than approx. −8°C), however, represent very rare events in our climate dataset (0.0106% of hours from 1 January 1990 to 31 December 2016, excluding August and September). Hence, negative spore death predictions were forced to zero. Model B was parameterized using a thermal performance curve of STB LP from Bernard *et al*. [29] (see electronic supplementary material; Material and methods; figure 3).

The thermal performance curve (equation (2.1)) for *Z. tritici* used in model B [29] describes a temperature-dependent LP for STB disease development, where the LP is defined as the time elapsed from inoculation until 37% of maximum sporulation (by area) had developed. Parameters for the thermal performance curve were set as the pooled estimates of three strains of *Z. tritici* as reported by Bernard *et al.* [29]: LP_min_ = 691 h, Curv = 20.4 h°C^−2^, *T*_opt_ = 18.4°C. (Note: Bernard *et al.* [29] define *T* as mean leaf temperature. Here, we assumed equivalency between mean leaf temperature and mean canopy temperature, as only the latter is available in our climate data.) From this thermal performance curve, we calculated relative rates for temperature-dependent LP. Similar to model A, we then modelled disease development risk using a cumulative Weibull distribution. For model B, because Bernard *et al.* [29] do not provide direct data for sporulation dynamics over time at *T*_opt_, we were unable to estimate *α* and *γ* directly. Instead, we first extracted data from Bernard *et al.* [29] for a *Z. tritici* leaf chlorosis/necrosis progression curve under optimal leaf temperature class (16.6–18.7°C) using DataThief III [33]. We approximated sporulation dynamics at *T*_opt_ from this curve. However, we shifted the sporulation curve by 94 h. This ensures consistency with equation (2.1), where 37% of maximum sporulation occurred under *T*_opt_ at LP_min_ (691 h) [29], and accounts for the likelihood of chlorosis occurring before tissue necrosis/pycnidia development [34]. We hence estimated the Weibull parameters as *α* = 822 and *γ* = 4.5; see electronic supplementary material, figure S5. Weibull parameters were chosen based on iterative fitting to available data, with the *α* and *γ* that resulted in the smallest residual sum of squares selected. The LP is assumed not to differ between ascospores and pycnidiospores in model B. The thermal performance curve of *Z. triticis* is given by

2.1 $$\mathrm{TPC}(T)=\frac{1}{LP_{\min}+\mathrm{Curv}\times {\left(T-T_{\mathrm{opt}}\right)}^{2}},$$

LP is latent period (hours post-inoculation, hpi); LP_min_, minimum latent period at *T*_opt_ (hpi); *T*, mean canopy temperature (°C); *T*_opt_, optimal mean leaf temperature (°C); hpi, hours post-inoculation. Note: originally, LP_min_ was in days post-inoculation (dpi) (28.8) and Curv was in dpi °C^−2^ (0.85) [29]. In model B, LP_min_ is in hpi (691) and Curv is in hpi °C^−2^ (20.4).

### (b) Model execution

We defined the period when infection is possible as 00.00 on 1 October to 23.00 on 31 July: from seed sowing to completion of grain filling for UK winter wheat (Zadok's growth stage g66, https://cereals.ahdb.org.uk). We downloaded historical weather estimates (canopy temperature (°C), canopy surface water content (g m^−2^), and total precipitation (mm)) for these months in the major wheat-growing regions of the UK (1.97° W to 1.97° E, 50.0 to 55.0° N) from 1990 to 2016, inclusive, at 3-hourly intervals and 0.5625° spatial resolution, from the Japanese 55-year reanalysis (JRA55) via the Research Data Archive of CISL (NARC) (https://rda.ucar.edu/datasets/ds628.0/) [30]. Canopy temperature and surface water content data were linearly interpolated, and total precipitation data divided by 3, to give hourly estimates for use in models A and B.

In models A and B, we calculated relative hourly ascospore cohort size using the ascospore influx rate equation in Kitchen *et al.* [35]: *X*(*t*) = *ηt*^2^e^−λ*t*^. In this equation, *η* is the ascospore influx coefficient (units = T^−3^) and *λ* is the ascospore influx decay rate (dimensionless) (electronic supplementary material, figure S6). While this equation expresses ascospore deposition as a function of degree-days, our models run in hours, where *t* is in hours (units =T). We chose the value of the parameter *λ* (*λ* = 0.00159) such that peak ascospore delivery occurs in late November, in line with spore-trapping data [36,37] and the role of ascospores as primary inoculum [38], maintaining the overall shape of the curve used by Kitchen *et al.* [35]. This decision carries two caveats. Firstly, that mapping degree-days to time obscures the second recorded peak of ascospore production around the time of harvest. Our model, however, is halted at the end of grain filling, so this second ascospore peak occurs outside of the times modelled and can be disregarded. Secondly, directly converting the degree-day curve into hours means that our models assume ascospore production to be independent of the weather parameters that are accounted for by the use of degree-days. This caveat is considered in the Discussion. The average temperature at the start of the model (October) is 11.25°C, giving an LP of approximately 71 days (1704 h) (equation (2.1); [29]). Hence, in our models pycnidiospores may arrive from 1 December, allowing two months for sporulating pycnidia to develop from ascospores that arrived at the beginning of the growing season. Further, pycnidiospores, since these are rain-splash dispersed, only arrive during precipitation. Because of the polycyclic nature of *Z. tritici* infection, we assumed that pycnidiospores were available during every precipitation event after 1 December. We did not attempt to model the dependency between infection levels and pycnidiospore cohort size, nor the relative size of each pycnidiospore cohort compared with the size of ascospore cohorts. This is because, to our knowledge, no data exist concerning the number of pycnidiospores produced per ascospore or per pycnidiospore infection, nor concerning the dependence of this on weather during infection and dispersal. Neither is it known whether *Z. tritici* exhibits density-dependent germination. The pycnidiospore cohort size was thus set arbitrarily to the same size as the largest possible ascospore cohort size. In model A, we assumed that germination and growth take place only if the canopy surface water content exceeds zero. An example spore germination trajectory is shown in electronic supplementary material, figure S7. In model B (equation (2.1)), surface and apoplastic growth cannot be separated; thus, the transition between spores and pycnidia in model B could be considered either canopy surface water-dependent, as for surface growth prior to penetration [7], or -independent, as for apoplastic colonization. Model B was thus run with and without this dependency, hereafter referred to as model B1 and B2, respectively. In model A, the total relative number of spores germinating per hour is the sum of all germinating cohorts of pycnidiospores/ascospores; the relative number of spores growing and thence penetrating stomata per hour is the sum of all growing cohorts of pycnidiospores/ascospores, and indicates relative infection risk. In model B, STB disease is indicated by the sum of all sporulating cohorts of pycnidiospores/ascospores.

### (c) Comparison of model output with observed STB disease data

Observed STB disease data (% field area affected by STB) from across the UK (2002–2016) were downloaded from the Agriculture and Horticulture Development Board (AHDB—available upon request, https://cereals.ahdb.org.uk). Eighty sites fell within the area included in the model and each provided at least 1 year's observed disease data (electronic supplementary material, figure S8). Wheat plants were not treated with fungicide at any location. Approximate latitude and longitude values were determined for each site. Where the site was at county level, the approximate centroid point of the county was used. To compare observed STB disease data with model predictions of percentage infection/STB disease, site locations were rescaled to the spatial scale of the climate data used in models A and B (0.5625° × 0.5625° resolution, equating to approx. 2000 km^2^). Correlation coefficients were calculated between mean observed percentage STB disease in each location and the infection/disease predictions from models A0, A1, A2, B1 and B2. We also calculated correlation coefficients between the temporal mean of the observed percentage STB disease in each pixel and model predictions from models A0, A1, A2, B1 and B2.

## 3. Results

**Figure 4.**
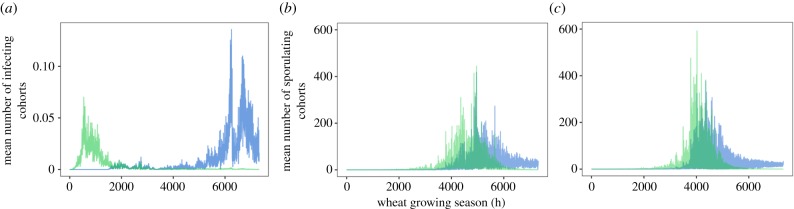
Average trajectory of *Z. tritici* infection (model A) and STB disease development (model B) over the wheat-growing season (1 October–31 July). (*a*) Model A0: infection refers to spore cohorts that germinate and grow hyphally along the leaf surface, leading to stomatal penetration. Models B1 (*b*) and B2 (*c*): disease development refers to spore cohorts that successfully infect and subsequently sporulate on the leaf surface. Infection/STB disease development is calculated for each hour of the growing season, as the mean of all pixels for all growing seasons in the climate dataset (winter 1990/summer 1991 to winter 2015/summer 2016). Green and blue lines represent (sexual) ascospores and (asexual) pycnidiospores, respectively.

In models A0–A2, ascospores largely infected wheat primarily at the beginning of the growing season, and pycnidiospores later in the growing season (figure 4*a*; electronic supplementary material, figure S4a, b). By contrast, in model B1 the trend of successful infection by both ascospores and pycnidiospores was largely positive across the growing season (figure 4*b*). In model B2, pycnidiospores followed a similar trend to model B1, but the majority of ascospores completed infection earlier in the growing season (figure 4*c*). Of note, neither model B1 nor B2 includes an explicit, wetness- or temperature-dependent cell death function, since this is assumed to be implicit in the length of the LP described by equation (2.1). However, this means that all spores in the model can overwinter and cause disease once favourable conditions return. By contrast, model A removes spores during all dry periods in a temperature-dependent manner. The size of the spore cohorts in model A thus declines in the absence of successful infection.

**Figure 5.**
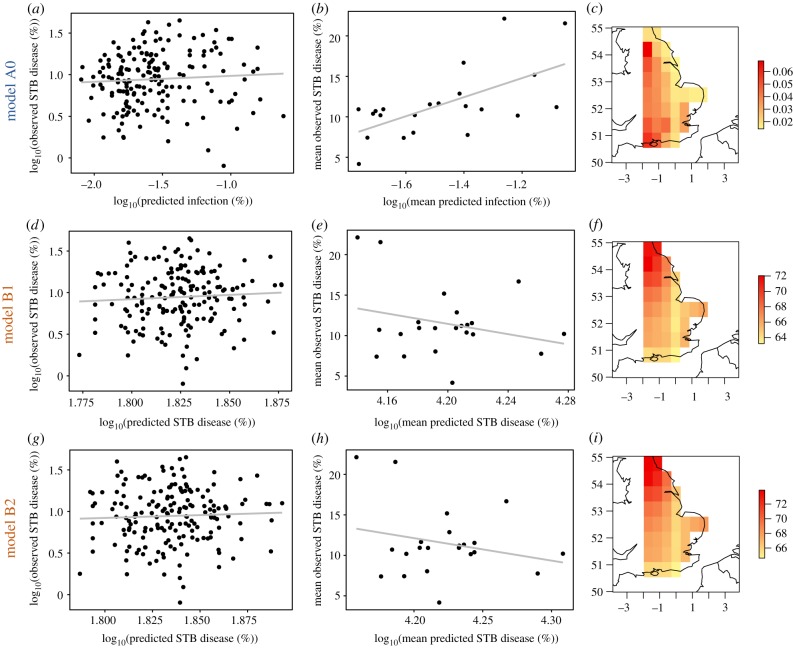
Pearson's correlation between model outputs and observed STB disease (%). Predicted infection (predicted STB disease) was calculated as the average total infection (average total STB disease) in a given pixel, for each growing season. Observed STB disease was calculated as the average disease of each farm, for each growing season. (*a*) Model A0, *r* = 0.065, *p* > 0.05. (*d*) Model B1, *r* = 0.065, *p* > 0.05. (*g*) Model B2, *r* = 0.043, *p* > 0.05. Mean predicted infection (mean predicted STB disease) was calculated as the average total infection (average total STB disease) in a given pixel, pooled for all growing seasons. Mean observed STB disease was calculated as the average disease of all farms located in a given pixel, pooled for all growing seasons. (*b*) Model A0, *r* = 0.623, *p* < 0.005. (*e*) Model B1, *r* = −0.261, *p* > 0.05. (*h*) Model B2, *r* = −0.236, *p* > 0.05. Mean predicted infection (%) across the spatial scale of model A0. (*f*,*i*) Mean predicted STB disease (%) across the spatial scale of models B1 (*f*) and B2 (*i*). Values of *x* and *y* refer to longitude and latitude, respectively. (*a*,*d*,*g*) *n* = 179; (*b*,*e*,*h*) *n* = 22. Growing seasons included in (*a*,*b*,*d*,*e*,*g*,*h*) are winter 2001/summer 2002 to winter 2015/summer 2016. Growing seasons included in (*c*,*f*,*i*) are winter 1990/summer 1991 to winter 2015/summer 2016. Data were log_10_-transformed prior to statistical analysis to improve fit to the underlying assumptions of Pearson's correlation test.

We compared model predictions of infection/STB disease with observed disease data. Infection/STB disease predictions from models A and B did not correlate significantly with percentage observed disease: (A0: *r* = 0.065, *p* > 0.05; A1 *r* = 0.089, *p* > 0.05; A2, *r* = 0.058, *p* > 0.05) (B1: *r* = 0.065, *p* > 0.05; B2: *r* = 0.043, *p* > 0.05; figure 5*a*,*d*,*g*; electronic supplementary material, figure S3a,d). However, when averaged for each pixel across the temporal scale of observations (2002–2016), infection risk predictions from models A0, A1 and A2 correlated significantly with temporal mean pixel percentage observed disease (A0: *r* = 0.623, *p* < 0.005, figure 5*b*,*c*; A1: *r* = 0.521, *p* < 0.05; A2: *r* = 0.646, *p* < 0.005, electronic supplementary material, figure S3b,d), while models B1 and B2 did not (B1: *r* = −0.261, *p* > 0.05; B2: *r* = −0.236, *p* > 0.05; figure 5*e*,*f*,*h*,*i*). Models A1 and A2 also showed no significant correlation between predicted infection risk and observed disease; A2 performed slightly better than model A0 with respect to predictions of temporal mean disease, but this was not significant (electronic supplementary material, figure S3).

## 4. Discussion

We present a new mechanistic model (A) for predicting weather-dependent Septoria tritici blotch disease. This model is parameterized with experimentally derived data, and predicts germination and growth of *Z. tritici* on the wheat leaf to estimate infection/STB disease. We compared model A's predictions with observed disease data and did the same for the predictions of a similar model (B) in which the experimentally parameterized portion of model A was replaced with a modified thermal performance disease equation from Bernard *et al.* [29]. In addition, we compared the temporally pooled results of each model with temporally pooled disease observations, to determine whether either model can identify areas of the UK where risk from STB disease has been particularly high or low over the modelled period.

Neither model A nor B showed significant correlation between predicted risk and observed disease in a given year (figure 5*a*,*d*,*g*). Moreover, model B showed no improvement when leaf wetness data were incorporated (figure 5*g*). Model A, however, successfully distinguished between areas with differences in disease risk over the whole modelled time period, while model B did not (figure 5*b*,*e,h*). Possible explanations for the failures in prediction by either model can be divided into limitations associated with the observed disease and climate data, and limitations of the model itself.

The observed disease data relate to 179 observations from 80 farms, and were averaged across the 22 pixels of approximately 0.5° × 0.5° for which climate data were available to drive the models [30]. Disease observations therefore include between-farm spatial variation, which is not captured by the models presented here. Spatial heterogeneity in disease severity may also occur even within a field owing to microclimate effects within the canopy, including differences between air temperature and leaf temperature [29]. It can be seen in electronic supplementary material, figure S9 that the per-pixel, observed disease data show a strong correlation between the mean and standard deviation. This indicates that in pixels where disease is common, there is more variation than can be explained by accounting for the differences between pixels. Thus, variations in climate between pixels may not be sufficient to capture all of the relevant within-pixel variability in disease. To overcome this, higher resolution data for climate-derived parameters, such as temperature, rainfall and leaf wetness, would be required. However, to capture very fine scale, even within-field variation, would require specialized recording equipment within wheat canopies—in other words, data collection beyond the scope of disease risk model development. Further, the observed disease data represent the percentage field area affected by STB, but not lesion area or pycnidiation density. By contrast, both models give infection/STB disease based on the percentage of spores landing on the leaf that progress to cause disease. It has been shown that, in the absence of other factors such as disease saturation, one spore is sufficient inoculum to form one lesion [39], and this is reflected in the field by the frequent dominance of a single genotype within lesions [40]. Therefore, the model output represents the percentage of spores progressing to lesions. There may, however, be heterogeneity in lesion formation, giving variable observed disease outcomes from the same percentage of infecting spores. Temporal heterogeneity is likely to be of particular importance in model A, which shows large variation in numbers of infections occurring at different points during the growing season (figure 4*a*). Such limitations in observed disease data could be overcome only with an intensive research effort to score lesions in the field.

For model A, it is clear that the largest errors in annual disease risk predictions occur where observed disease is very high and predicted risk is very low. This pattern cannot be explained by the limitations of the observed data, which apply to all risk predictions, but is likely due to the limitations of model A itself. The most obvious limitation of model A is that, in contrast to previous models (e.g. [24,25]), model A does not contain any host growth parameters nor data concerning the resistance status of the wheat cultivars planted. This omission means that unexpectedly high disease may occur where a variety of wheat with low STB resistance has been planted, or where the constantly evolving pathogen breaks host resistance in a particular wheat cultivar [41,42]. Previous models (e.g. [24,25]) specifically consider wheat growth stage as a proxy for leaf availability for infection and lesion development, while model A assumes that if a spore can grow, it can cause a lesion. However, saturation of lesion formation following leaf inoculation with increasing numbers of spores has been demonstrated [39,43]. Depending on disease progression and the timing of disease observation, this may cause model A to over- or underestimate disease risk. Other environmental factors that may be of relevance to disease progression, such as agronomic factors and atmospheric composition, have not been considered in this work. These may be required for a fully predictive model [44]. It is important to note that by converting the degree-day curve for ascospore delivery [35] into hours, model A fails to account for the dependency of ascospore production and delivery upon the climatic variables that relate degree-days to time. Despite this, it is generally accepted that ascospore deposition on winter wheat in the Northern Hemisphere peaks in late autumn and tails off throughout the rest of the period of wheat cultivation covered by model A [35–37]. Further, it must be noted that, in the absence of data allowing us to model the weather-dependent rate of pycnidiospore production per infection event (either asco- or pycnidiospore initiated), we have arbitrarily designated the size of every pycnidiospore cohort as equal to the largest possible ascospore cohort size. This may be either an over- or underestimate at different times within the growing season. In theory, it is possible for every infection to generate multiple pycnidia, each holding at least 300 pycnidiospores [4], making a large underestimate theoretically possible. However, this underestimate is likely offset by infection saturation of leaves [39], density-dependent germination rates and loss of spores from the system (e.g. by rain-splash onto non-wheat surfaces) for which no data exist at present. These limitations apply equally to model B.

A second limitation of models A and B is that they assume a stable *Z. tritici* population whose responses to temperature and other parameters are the same as those of the experimental strains used. For model A, this is IPO323 (except for growth parameters in A2), while model B uses pooled data from three French field isolates [29]. In reality, multiple strains of *Z. tritici* co-infect in the field [40,41] and these may vary in their rate of progression through the disease cycle, lesion size and lesion clustering [45], all of which may affect observed percentage diseased leaves. *Zymoseptoria tritici* strains can also compete or co-operate on and in the leaf [46]. Strain IPO323 has not been compared with, and may not be representative of, UK field isolates from the modelled years. Our *in vitro* experiments showed cardinal temperatures (CTs) for *Z. tritici* strain IPO323 of 19.1 and 23.0°C for germination and growth, respectively. We note that these temperature optima are both high in comparison with UK daily mean temperatures (electronic supplementary material, figure S10), as well as being higher than the optimal temperature of equation (2.1) [29], which together may explain some cases of under-prediction by model A1. Further to this, recent work by Boixel *et al.* [32] investigated the CTs for growth in IPO323 and a collection of French *Z. tritici* field isolates. We noted that this work demonstrated that IPO323 was a clear outlier compared with the field isolates, showing an elevated (+5.5°C) optimal temperature. To investigate the importance of these findings for model A, we reran model A0, using the average CTs reported for the French field isolates [32] (model A2, electronic supplementary material, figure S3). This change neither altered the overall outcome of the comparison between observed and modelled disease (A0, *r* = 0.065 versus A2, *r* = 0.058), nor significantly improved the fit of the temporally pooled data (A0, *r* = 0.623 versus A2, *r* = 0.646). This result suggests that the disparity between optimal growth temperatures for IPO323 and the field isolates does not account for a significant part of the error in model A0—although data from UK field isolates are still lacking. Further, models A0–A2 are parameterized by the same IPO323 thermal death estimates (figure 2*c*); exploration of temperature-dependent death for additional field isolates under wet/dry conditions would clarify whether this contributed to error in model A. Finally, Suffert & Thompson [47] show that LP varies between populations and subpopulations of *Z. tritici*, pycnidiospores and ascospores, and is influenced by wheat growth stage. Although such sources of variability were not accounted for in our models, should appropriate data become available it would be interesting to test their effect upon the utility of each model.

Model B has two further limitations, which likely contribute to its failure to predict not only annual disease risk, but also time-averaged risk per pixel, where model A performs well. Firstly, equation (2.1) [29], which underpins model B, is parameterized using an experimentally derived thermal performance curve for *Z. tritici,* but the underlying experimental data are restricted to the range 10–22°C. As this does not cover the entire range of conditions experienced during the UK wheat-growing season, we were obliged to extrapolate this curve for model B. By contrast, the data that we have collected to parameterize model A span 0–30°C and require a much smaller degree of extrapolation (figure 2*a,b*). Secondly, the equation in model B has no scope for incorporating the death of spores over time. Thus, any spore that arrives on the leaf under unfavourable conditions simply remains in the model until it is able to grow and infect. Further, Bernard *et al.* [29] show a reduction in maximum disease at sub-optimal temperatures. Equation (2.1) does not account for this reduction and there are no data available with which to explicitly apply this reduction to predicted disease under changing temperature conditions. These limitations account for the stark differences between model A and models B1 and B2 seen in figures 4 and 5: in model A, spores die rapidly in dry periods and are removed from the model, whereas in B1 they are merely paused and in B2 suffer no effect. Model A shows successful infections only under permissive temperature and wetness, giving peaks of infection in autumn and in spring/summer (figure 4*a*). Both iterations of model B show rapid disease saturation (figure 4*b*,*c*), with very high percentages of spores completing infection (around 75%, when conditions are optimal, compared with 0.2% in model A0; figure 5*a*,*d*,*g*). Fones & Gurr [4] estimated that up to 10^11^ *Z. tritici* pycnidiospores can be produced per hectare of 5 × 10^6^ leaves. If 75% of spores lead to lesions, as in model B, we would therefore see up to 15 000 lesions per leaf under optimal conditions. By contrast, the approximate 0.2% success rate of model A under optimal conditions would give around 40 lesions per leaf. Each lesion may contain hundreds of pycnidia. These calculations indicate that a large overall overestimate of disease pressure is inherent to model B, likely as a result of the lack of temperature-dependent spore death during periods when weather prevents infection. This demonstrates the value of the experimentally derived data concerning temperature-dependent death that we incorporated into model A.

This demonstration, along with the comparative success of model A over model B, clearly shows that the use of experimentally derived parameters for biological processes in this mechanistic model leads to improved applicability (figure 5*b*). Such experimentally derived parameterization could be used for other crop pathogen models, where CTs, growth and death rates can be determined *in vitro.* For many plant pathogens, relationships between climate parameters and disease are simpler [2,20]. The need to describe additional epiphytic persistence and growth for *Z. tritici*, however, makes this pathogen particularly amenable to our experimentally parameterized, mechanistic approach. Other plant pathogens that persist epiphytically include many important bacteria (e.g. *Pseudomonas syringae* [48]). Our approach may also be useful for certain climate-responsive animal pathogens. White-nose syndrome in bats, for example, is caused by the fungus *Pseudogymnoascus destructans*, which is sensitive to both temperature and humidity in bat hibernacula [42,49]. Further, insect-vectored diseases such as malaria and dengue fever are susceptible to mechanistic modelling of their response to climate change, with experimental parameters applying to the vectors as well as to the disease-causing organisms. Malaria rates have been shown to be related to temperature and to require a threshold level of rainfall, as these factors influence the mosquito vectors [50,51], while dengue fever rates are related to temperature through the effect of warmth on viral replication rates [50]. The use of mechanistic models in human disease spread modelling has been increasing rapidly [51]. Interestingly, the use of unimodal response distributions to determine CTs for processes such as growth and germination, as we have done here, though common in plant disease models, is relatively new in the areas of animal and human disease modelling—for instance, this approach was first applied to malaria models in 2013 [51–53].

Despite the demonstrable advantages of the mechanistic model, model A cannot predict annual disease risk from STB (figure 5*a*). This indicates that at least a portion of the intractability of STB to disease modelling, hypothesized to be due to the effect of poorly understood, complex interactions between multiple factors such as light, temperature and humidity [18,54], remains. We propose that a mechanistic model based on a thorough understanding of the pathogen's infection biology should eventually be incorporated, alongside agricultural parameters such a frequency of fungicide applications, into a wider model that includes host parameters (e.g. that developed by Robert *et al.* [27]). We note that, while the disease observations used here relate to fungicide-free wheat growth, fungicide use and subsequent evolution of fungicide resistance in the pathogen is an important consideration in disease management. Thus, fungicide usage and pathogen responses must be considered alongside other pathogen features such as variation in CTs [53,55] and infection biology [46], as well as host features such as resistance status, in any comprehensive, mechanistic STB model. Only a mechanistic model that considers all aspects of the disease triangle—host, pathogen and environment—is ever likely to achieve significant predictive power for STB.

## Supplementary Material

Supplementary Materials & Methods

## Supplementary Material

Data

## Supplementary Material

Figure S1

## Supplementary Material

Figure S2

## Supplementary Material

Figure S3

## Supplementary Material

Figure S4

## Supplementary Material

Figure S5

## Supplementary Material

Figure S6

## Supplementary Material

Figure S7

## Supplementary Material

Figure S8

## Supplementary Material

Figure S9

## Supplementary Material

Figure S10

## Supplementary Material

Figure S11

## Supplementary Material

Supplementary Figure captions

## Data accessibility

All data and code associated with this work are available as supplementary material or on Github (https://github.com/thomaschaloner/A_new_mechanistic_model_of_weather-dependent_Septoria_tritici_blotch_disease_risk), respectively.
