# Supplementary material for: A new mechanistic model of weather-dependent Septoria tritici blotch disease risk
Source: Philos Trans R Soc Lond B Biol Sci. 2019 May 6;374(1775):20180266. doi: 10.1098/rstb.2018.0266 (PMC6553599; doi:10.1098/rstb.2018.0266)
Supplement: Supplementary Materials & Methods [file rstb20180266supp1.docx]

**Supplementary Materials & Methods**

***Z. tritici* strains and experimental conditions**

All experiments were conducted using *Zymoseptoria tritici* IPO323 or a variant expressing cytoplasmic eGFP [^[[1]](#endnote-1)^]. *Z. tritici* was cultured on Yeast-Dextrose-Peptone (YPD) agar at 18 ^o^C. All experiments were conducted twice independently, unless otherwise specified.

**Preparation of leaf wax slides**

One gram of wheat leaf was submerged for 5 minutes in 10 mL chloroform with shaking. Five sequential 100 μL aliquots of the resulting solution were pipetted onto hydrophobic glass slides, allowing the chloroform to evaporate. Slides were left to dry for at least 1 h before inoculation with *Z. tritici*.

***Z. tritici* temperature-dependent germination rate**

IPO323-eGFP blastospores were suspended in dH_2_O at 1x10^5^ cfu/mL. 100 μL was added to each leaf wax slide and incubated inside moist chambers, for 24 h at 3, 8, 11, 14, 17, 19, 20, 23, 26, 28, or 31 ^o^C. At 24 h, spores were observed by confocal microscopy at 40 x magnification. A minimum of 50 individuals were scored in each of 3-5 fields of view, on 3 independent slides per temperature. Hyphae per cell were enumerated for each live (fluorescent) individual.

***Z. tritici* temperature-dependent *in vitro* growth rate**

Fifty microliters of IPO323-eGFP blastospore suspension at 1x10^7^ cfu/mL were added to 10 mL of 1/10 YPD broth. Duplicate cultures were maintained at 0, 5, 9, 15, 18, 20, 25, 28, and 30 ^o^C. At various time points (days 0-6, 8, 10 and 15, plus days 7 and 9 for cultures maintained at 18, 20, and 25 ^o^C), 50 μL of culture was imaged by confocal microscopy. Ten 11 μm Z-stacks per slide were captured at random xy co-ordinates. Images were thresholded to distinguish gfp-fluorescence from background and % image area containing fluorescence used as a proxy for biomass. Growth rate was estimated as the slope of the linear regression between square root(cytoplasmic fluorescence) and day; see Figure S11.

***Z. tritici* temperature- and wetness-dependent thermal death rate**

For ‘wet’ incubation conditions, leaf wax slides were inoculated with 100 μL 10^7^ cfu/mL IPO323 spore suspension, and incubated for 4 h at -3, 0, 5, 9, 15, 20, 25 or 30 ^o^C in moist chambers. Suspensions were made with dH_2_O at incubation temperature. Propidium iodide (PI; 20 μL, 1 mg/L) was added and spores imaged immediately by confocal microscopy. For ‘dry’ incubations, the same procedure was followed except inoculated slides were dried at 18 ^o^C for 2 h before 4 h incubation, with no moist chamber. PI was added with 100 μL preheated dH_2_O, to give the same stain concentration as for wet cells. For each slide, 3-6 Z-stacks were captured at random xy positions (min. 22 spores per image, mean = 118). Cells were scored as alive if PI fluorescence was restricted to the extracellular membrane, or dead if any internal fluorescence was observed (including nuclei). Three wet and three dry slides were stained and imaged without the 4 h incubation to determine the baseline proportion dead cells in a freshly prepared blastospore suspension. The relationship between temperature and the per-hour increase in proportion dead cells under wet and dry conditions was estimated by linear regression.

1. Kilaru S, Steinberg G. Yeast recombination-based cloning as an efficient way of constructing vectors for Zymoseptoria tritici. Fungal Genetics and Biology. 2015 Jun 1;79:76-83. [↑](#endnote-ref-1)
