# Supplementary material for: A new mechanistic model of weather-dependent Septoria tritici blotch disease risk
Source: Philos Trans R Soc Lond B Biol Sci. 2019 May 6;374(1775):20180266. doi: 10.1098/rstb.2018.0266 (PMC6553599; doi:10.1098/rstb.2018.0266)
Supplement: Figure S1 [file rstb20180266supp3.pdf]

Model A

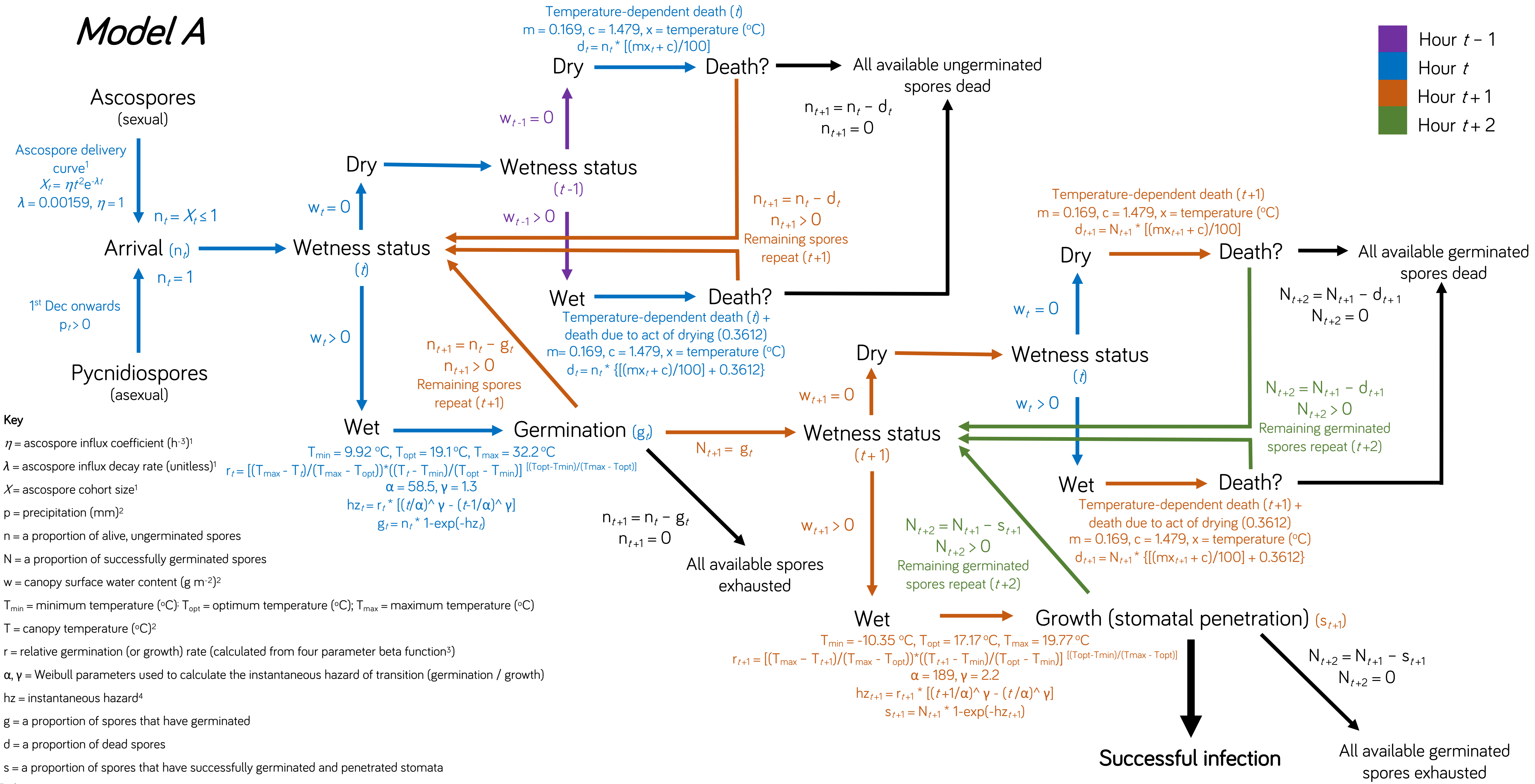

Key

η = ascospore influx coefficient (h^{-3})[1]

λ = ascospore influx decay rate (unitless)[1]

X = ascospore cohort size[1]

p = precipitation (mm)[2]

n = a proportion of alive, ungerminated spores

N = a proportion of successfully germinated spores

w = canopy surface water content ($g\ m^{-2}$)[2]

T_{min} = minimum temperature (°C); T_{opt} = optimum temperature (°C); T_{max} = maximum temperature (°C)

T = canopy temperature (°C)[2]

r = relative germination (or growth) rate (calculated from four parameter beta function[3])

α, γ = Weibull parameters used to calculate the instantaneous hazard of transition (germination / growth)

hz = instantaneous hazard[4]

g = a proportion of spores that have germinated

d = a proportion of dead spores

s = a proportion of spores that have successfully germinated and penetrated stomata

References

[1] Kitchen JL, van den Bosch F, Paveley ND, Helps J, van den Berg F. The evolution of fungicide resistance resulting from combinations of foliar-acting systemic seed treatments and foliar-applied fungicides: a modeling analysis. PloS one. 2016 Aug 29;11(8):e0161887

[2] Kobayashi S et al. 2015 The JRA-55 reanalysis: general specifications and basic characteristics. J. Meteorol. Soc. Jpn Ser. II 93, 5 – 48. (doi:10.2151/ jmsj.2015-001)

[3] Yan W, Hunt LA (1999) An equation for modelling the temperature response of plants using only the cardinal temperatures. Ann Bot 84:607-614

[4] Bebber DP, Castillo ÁD, Gurr SJ. Modelling coffee leaf rust risk in Colombia with climate reanalysis data. Philosophical Transactions of the Royal Society B: Biological Sciences. 2016 Dec 5;371(1709):20150458.
