# Supplementary material for: A new mechanistic model of weather-dependent Septoria tritici blotch disease risk
Source: Philos Trans R Soc Lond B Biol Sci. 2019 May 6;374(1775):20180266. doi: 10.1098/rstb.2018.0266 (PMC6553599; doi:10.1098/rstb.2018.0266)
Supplement: Figure S2 [file rstb20180266supp4.pdf]

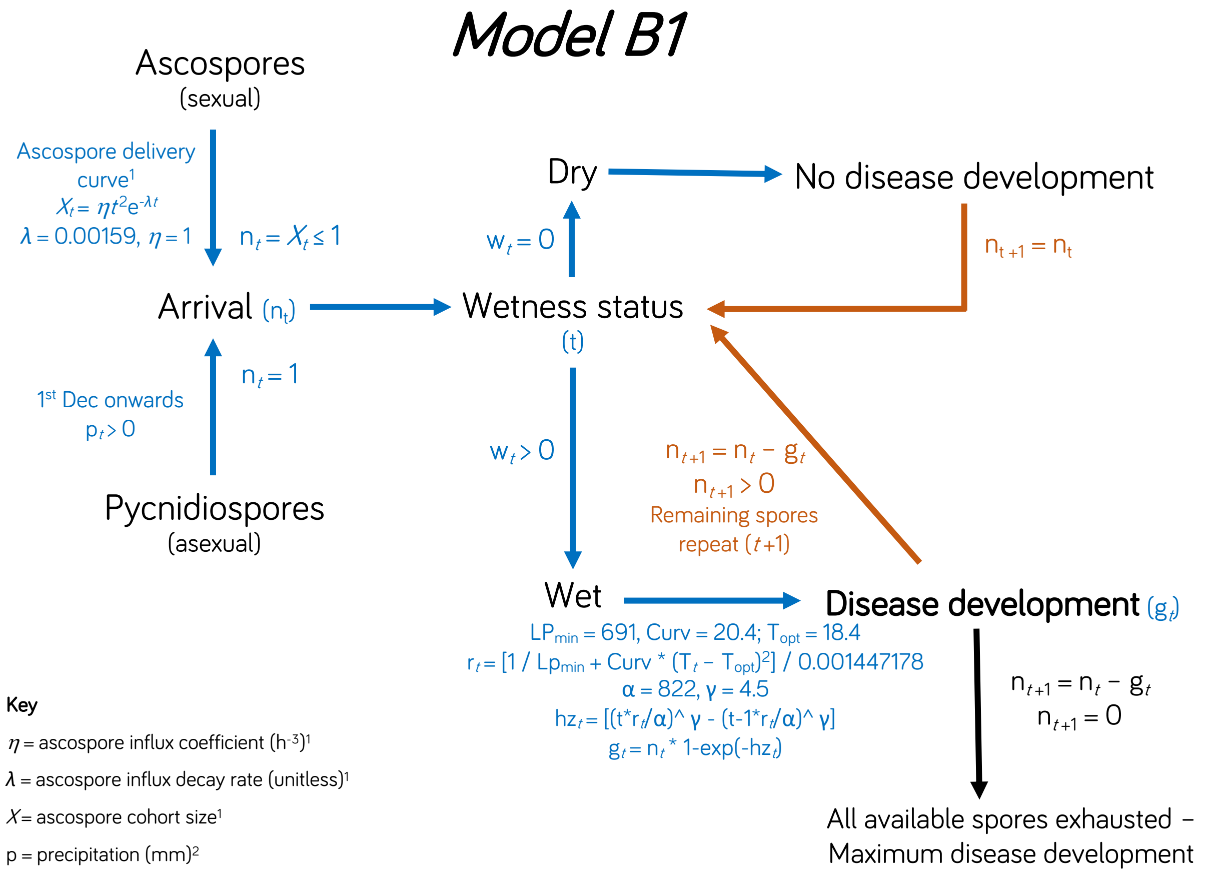

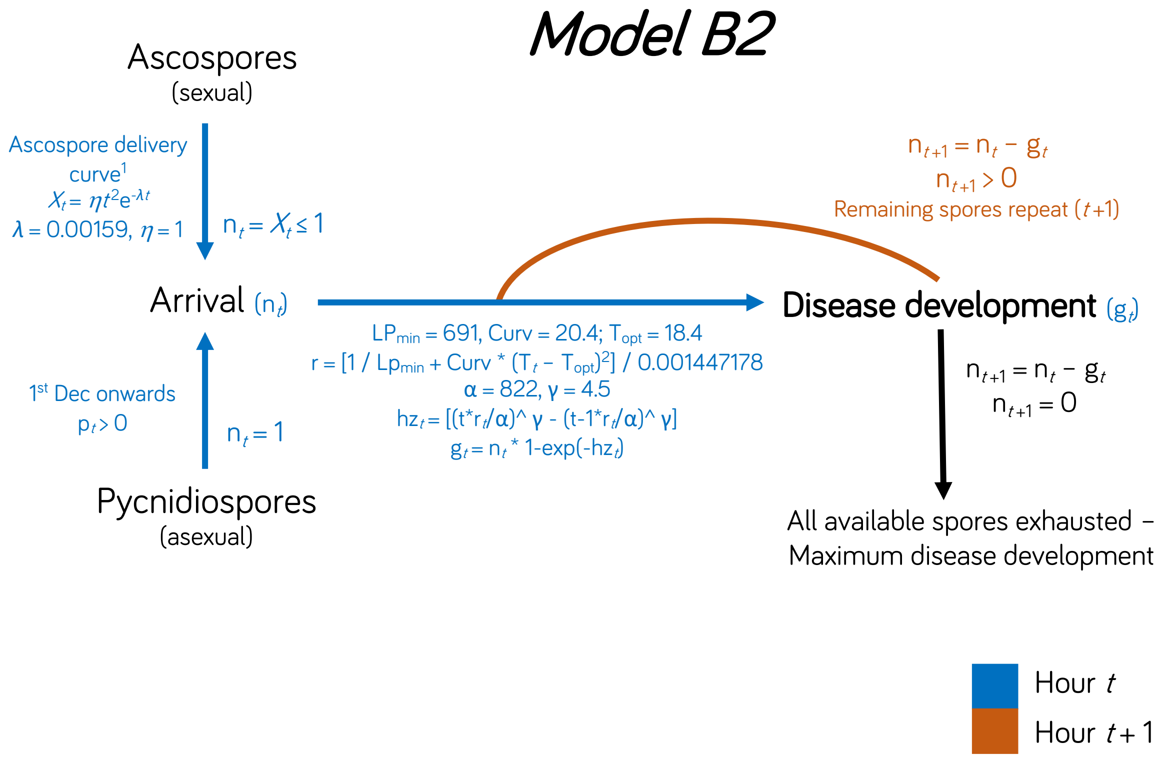

Key

η = ascospore influx coefficient (h^{-3})[1]

λ = ascospore influx decay rate (unitless)[1]

X = ascospore cohort size[1]

p = precipitation (mm)[2]

n = a proportion of spores available to cause disease

w = canopy surface water content ($g\ m^{-2}$)[2]

Lp_{min} = minimum latent period at T_{opt} (h); Curv = scaling parameter (hours post inoculation $^oC^{-2}$) ; T_{opt} = optimum temperature (^oC)[3]

T = canopy temperature (^oC)[2]

r = relative disease development rate (calculated by *Z. tritici* disease development thermal performance curve)[3]

α, γ = Weibull parameters used calculate the instantaneous hazard (hz) of transition (disease development)

hz = instantaneous hazard[4]

g = a proportion of spores that have successfully caused disease

Note: 0.001447178 = the absolute maximum rate of maximal disease development per hour at optimum temperature[2]. Dividing by 0.001447178 rescales rate to be bounded between 0 and 1, enabling relative rates od disease development to be calculated.

References

[1] Kitchen JL, van den Bosch F, Paveley ND, Helps J, van den Berg F. The evolution of fungicide resistance resulting from combinations of foliar-acting systemic seed treatments and foliar-applied fungicides: a modeling analysis. PloS one. 2016 Aug 29;11(8):e0161887

[2] Kobayashi S et al. 2015 The JRA-55 reanalysis: general specifications and basic characteristics. J. Meteorol. Soc. Jpn Ser. II 93, 5 – 48. (doi:10.2151/ jmsj.2015-001)

[3] Bernard F, Sache I, Suffert F, Chelle M. The development of a foliar fungal pathogen does react to leaf temperature! New Phytologist. 2013 Apr;198(1):232–40

[4] Bebber DP, Castillo ÁD, Gurr SJ. Modelling coffee leaf rust risk in Colombia with climate reanalysis data. Philosophical Transactions of the Royal Society B: Biological Sciences. 2016 Dec 5;371(1709):20150458.
