# Supplementary figures and images for: A new mechanistic model of weather-dependent Septoria tritici blotch disease risk
Source: Philos Trans R Soc Lond B Biol Sci. 2019 May 6;374(1775):20180266. doi: 10.1098/rstb.2018.0266 (PMC6553599; doi:10.1098/rstb.2018.0266)

Model A1

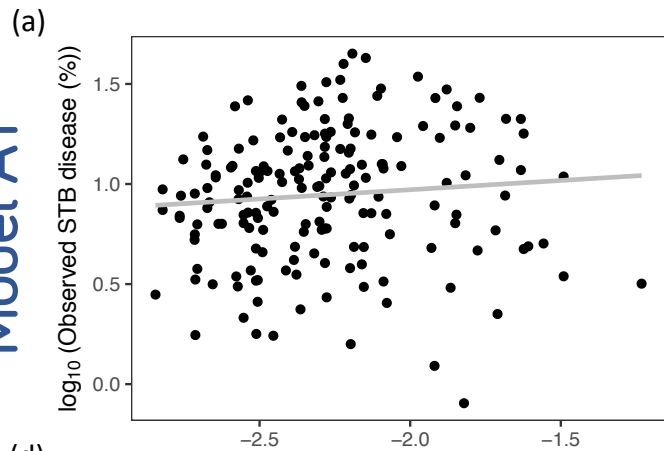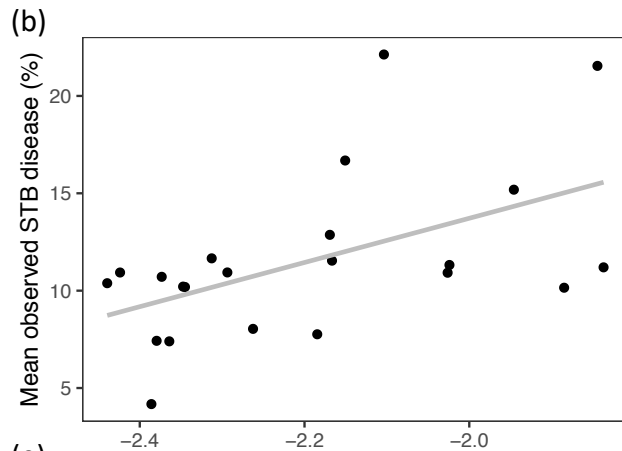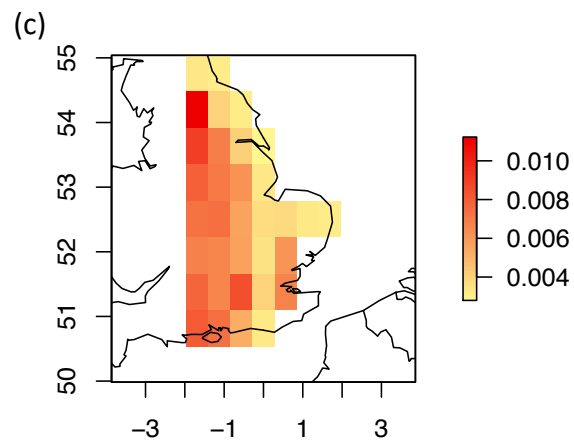

Model A2

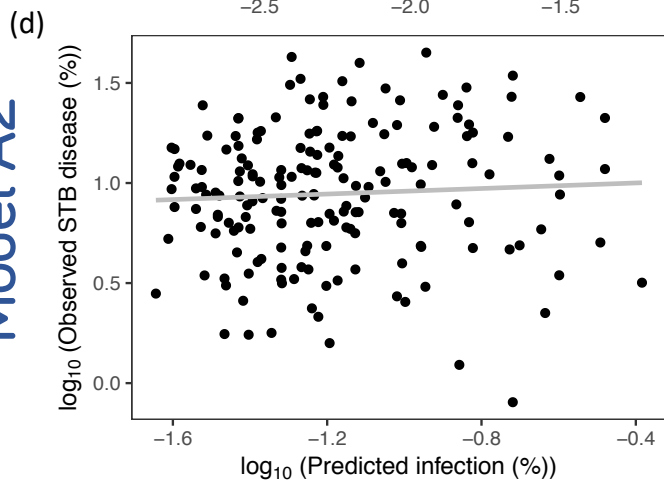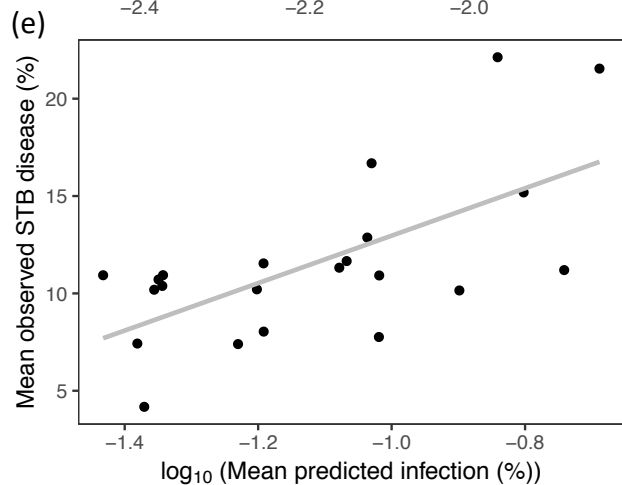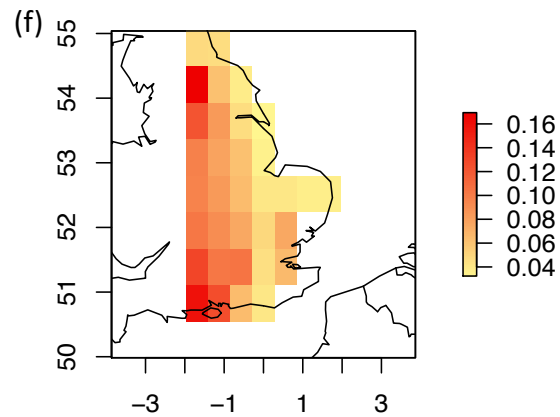

Supplement: Figure S3 [file rstb20180266supp5.pdf]

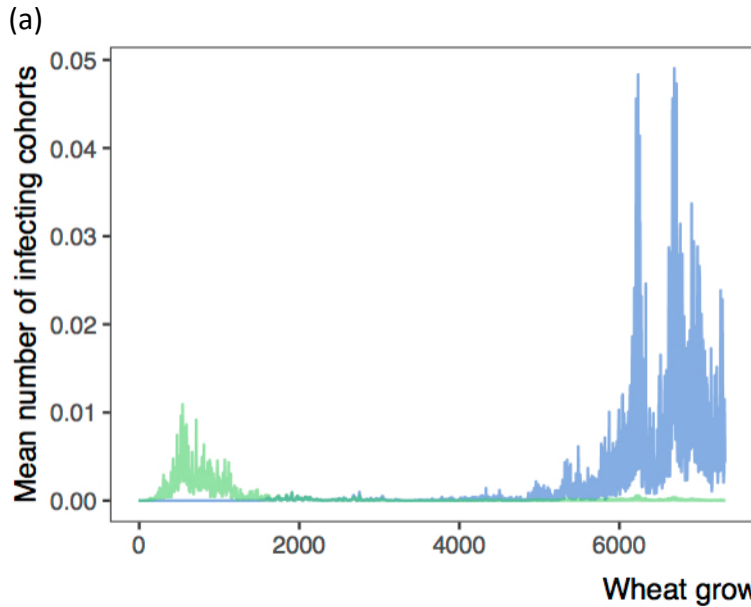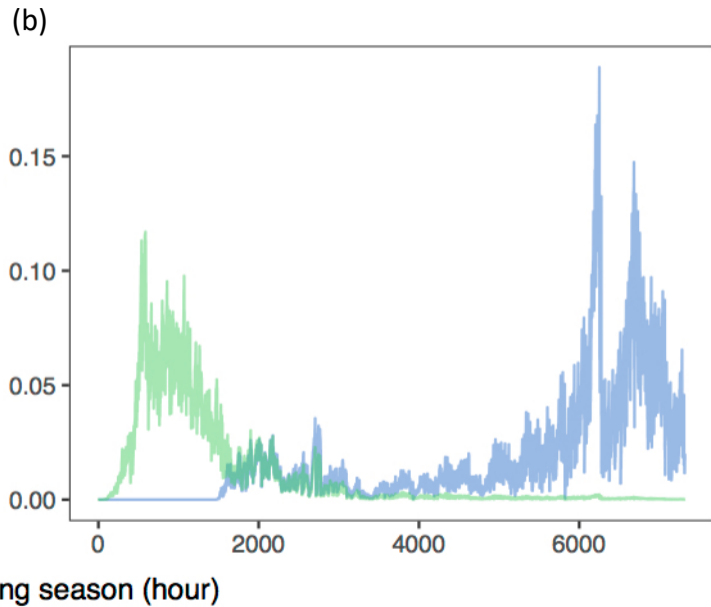

Supplement: Figure S4 [file rstb20180266supp6.pdf]

(a)  $\alpha = 58.5$ ,  $\gamma = 1.3$

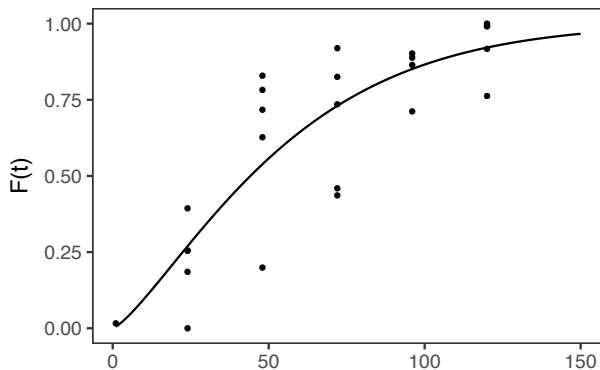

(b)

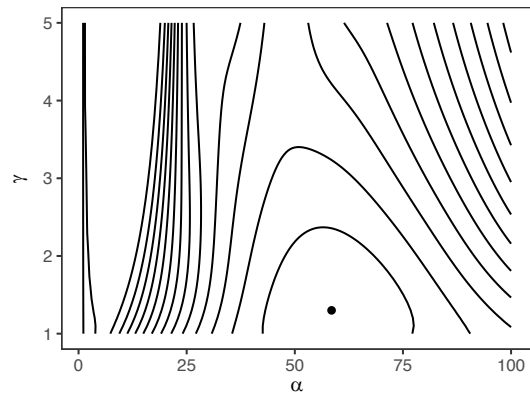

(c)  $\alpha = 189$ ,  $\gamma = 2.2$

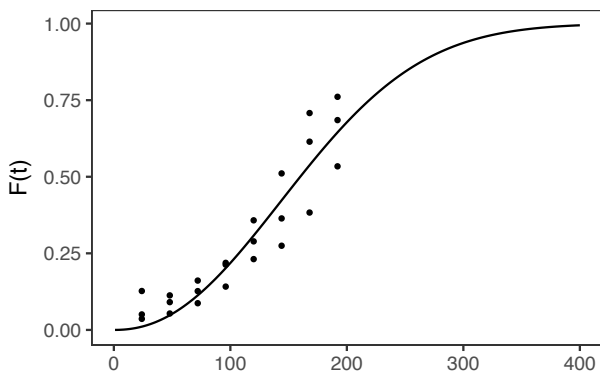

(d)

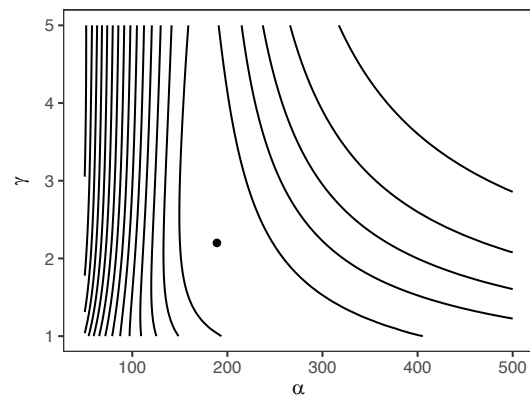

(e)  $\alpha = 822$ ,  $\gamma = 4.5$

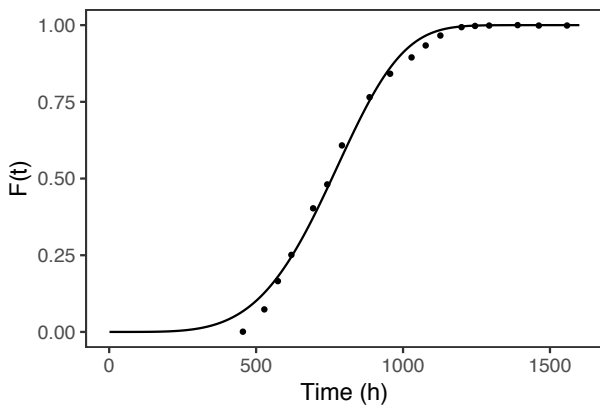

(f)

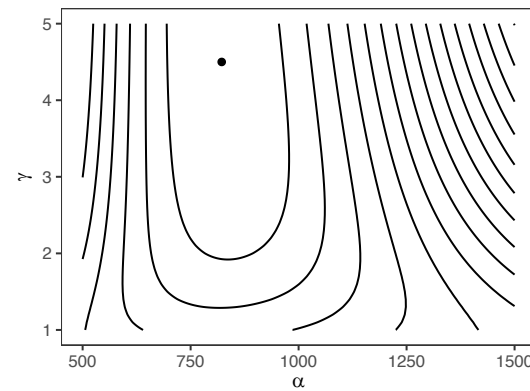

Supplement: Figure S5 [file rstb20180266supp7.pdf]

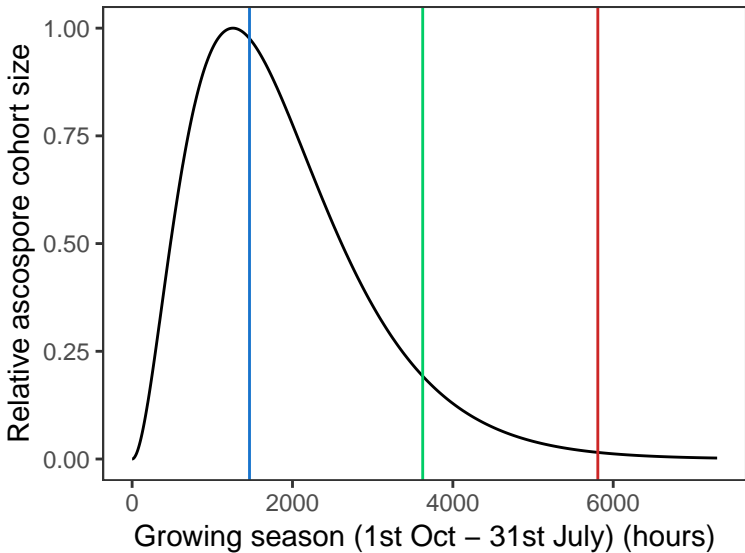

Supplement: Figure S6 [file rstb20180266supp8.pdf]

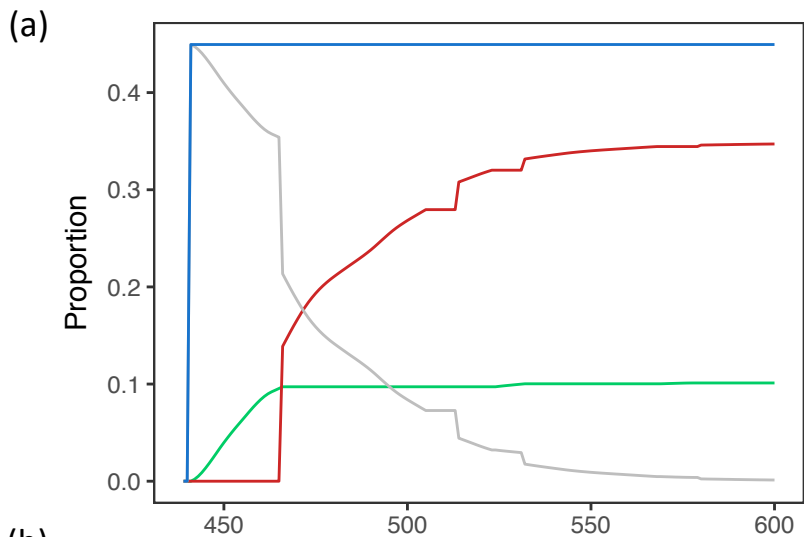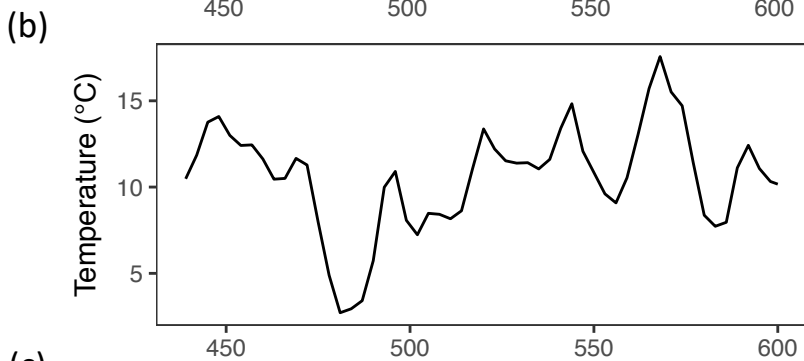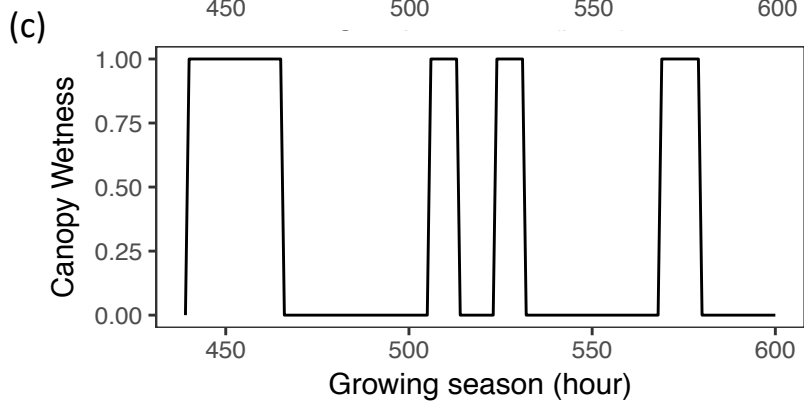

Supplement: Figure S7 [file rstb20180266supp9.pdf]

(a)

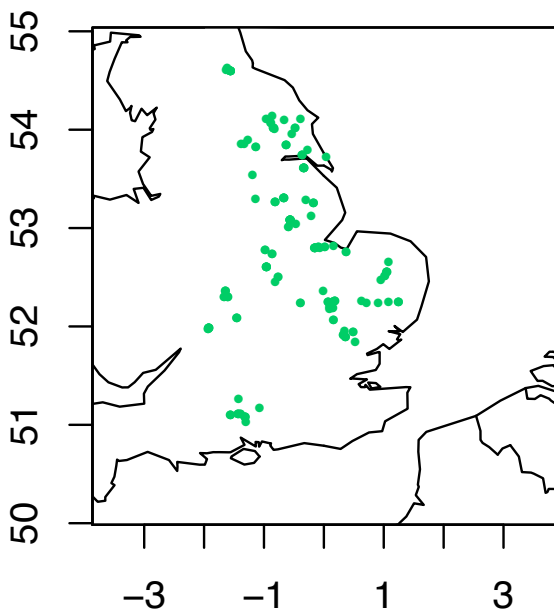

(b)

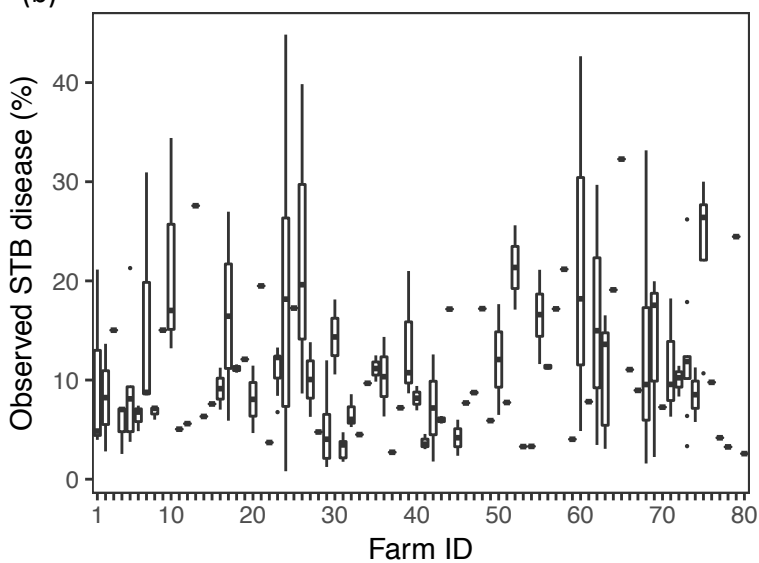

(c)

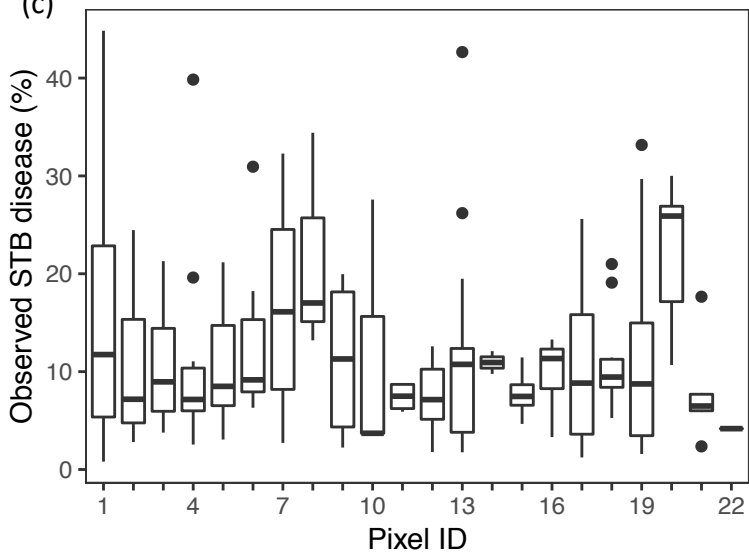

Supplement: Figure S8 [file rstb20180266supp10.pdf]

(a)

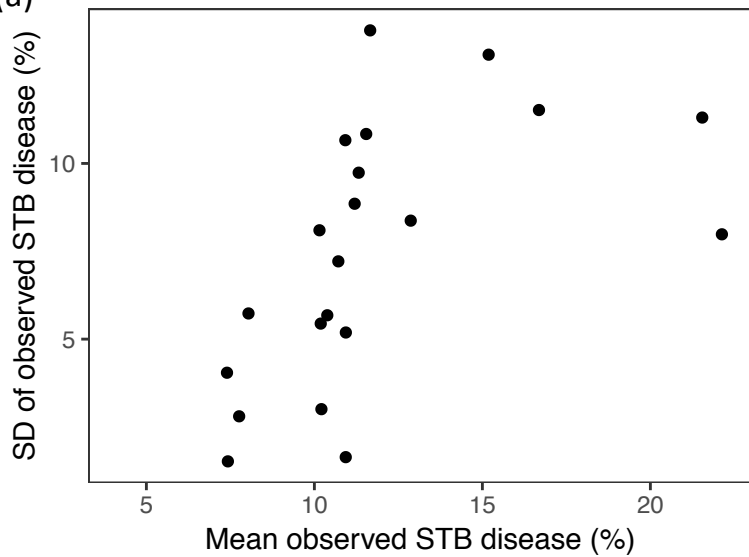

(b)

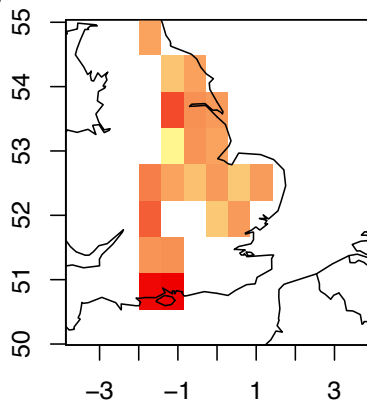

(c)

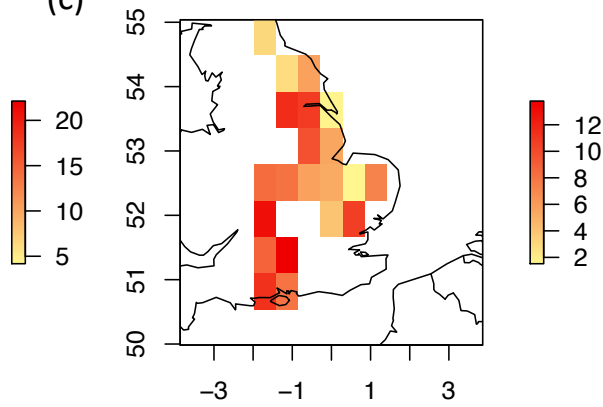

Supplement: Figure S9 [file rstb20180266supp11.pdf]

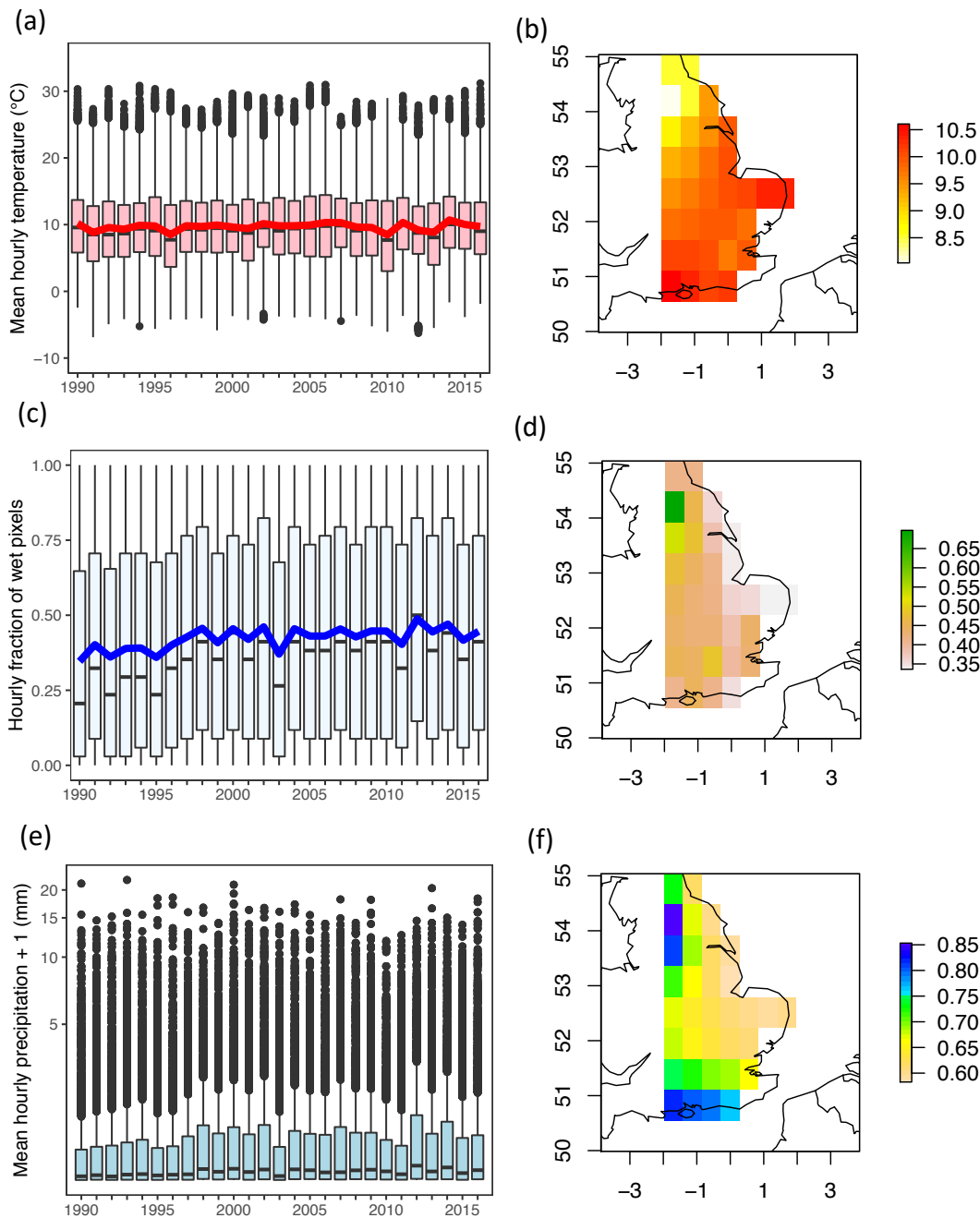

Supplement: Figure S10 [file rstb20180266supp12.pdf]

0°C

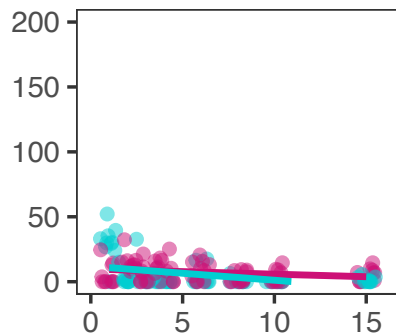

5

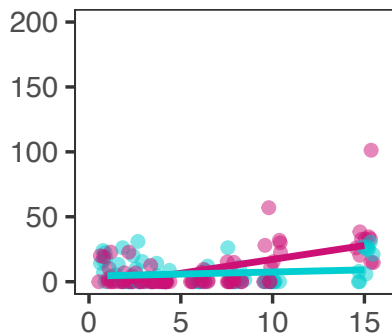

9

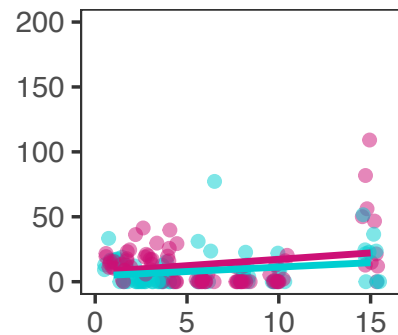

15

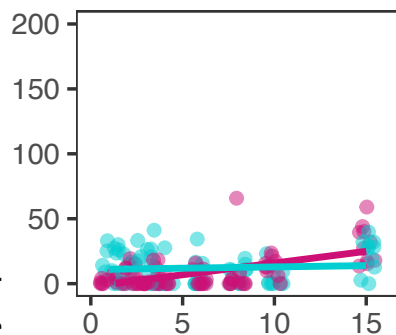

18

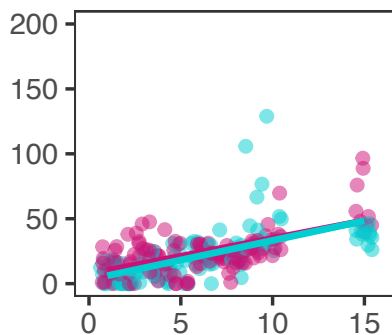

20

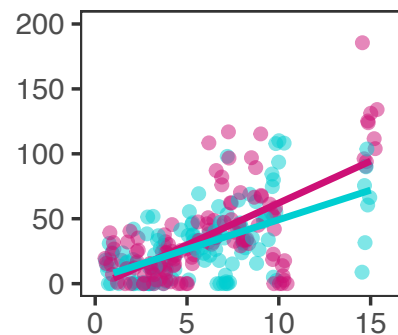

25

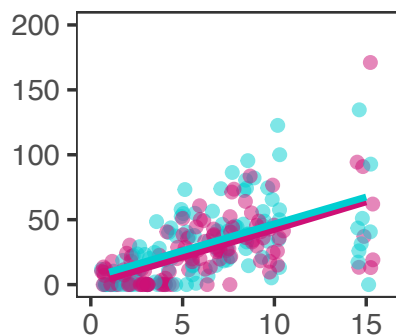

28

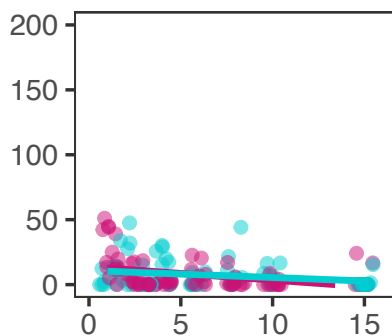

30°C

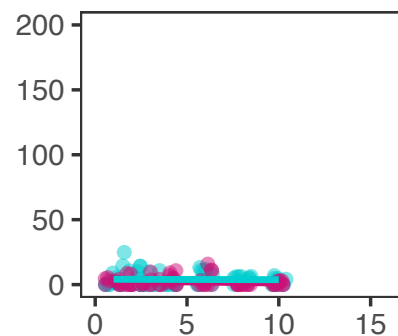 $\sqrt{\text{Cytoplasmic fluorescence}}$ 

days

Supplement: Figure S11 [file rstb20180266supp13.pdf]
