# Supplementary material for: A new mechanistic model of weather-dependent Septoria tritici blotch disease risk
Source: Philos Trans R Soc Lond B Biol Sci. 2019 May 6;374(1775):20180266. doi: 10.1098/rstb.2018.0266 (PMC6553599; doi:10.1098/rstb.2018.0266)
Supplement: Supplementary Figure captions [file rstb20180266supp14.docx]

**Supplementary Figure 1. Detailed schematic of model A.** Model A0, A1 and A2 are described by experimentally-derived parameters for the temperature- and leaf wetness-driven transition probabilities of spore germination; spore death; and hyphal growth, resulting in successful stomatal penetration (infection). Coloured arrows refer to specific hours. For example, all events defined by orange arrows take place at hour *t* + 1. Black arrows refer to either successful infection or exhaustion of available spores. T_min_, T_opt_ and T_max_ values refer to model A0; all other parameters are the same across models A0, A1 and A2. Details of each parameter, including units, are provided in the key. Model A was driven from 1^st^ October – 31^st^ July, for all pixels, for all growing seasons in the climate dataset (Winter 1990/Summer 1991 to Winter 2015/Summer 2016).

**Supplementary Figure 2. Detailed schematic of models B1 and B2.** Model B1 is described by the temperature- and leaf wetness-driven transition probabilities between spores landing on the leaf surface and pycnidiation; model B2 is only described by temperature. Coloured arrows refer to specific hours. For example, all events defined by orange arrows take place at hour *t* + 1. Black arrows refer to either successful disease development or exhaustion of available spores. Details of each parameter, including units, are provided in the key. Models B1 and B2 were driven from 1^st^ October – 31^st^ July, for all pixels, for all growing seasons in the climate dataset (Winter 1990/Summer 1991 to Winter 2015/Summer 2016).

**Supplementary Figure 3. Pearson’s correlation of model outputs against observed STB disease (%).** Predicted infection calculated as the average total infection in a given pixel, for each growing season. Observed STB disease is calculated as the average disease of all farms located in a given pixel, for each growing season. (a) Model A1, r = 0.089, p > 0.05. (d) Model A2, r = 0.058, p > 0.05. Mean predicted infection calculated as the average total infection in a given pixel, pooled for all growing seasons. Mean observed STB disease is calculated as the average disease of all farms located in a given pixel, pooled for all growing seasons. (b) Model A1, r = 0.521, p < 0.05. (e) Model A2, r = 0.646, p < 0.005. (c, f) Mean predicted infection (%) across the spatial scale of model A1 and A2. x and y values refer to longitude and latitude, respectively. (a, d) n = 179; (b, e) n = 22. Growing seasons included in (a, b, d, e) are Winter 2001/Summer 2002 to Winter 2015/Summer 2016. Growing seasons included in (c, f) are Winter 1990/Summer 1991 to Winter 2015/Summer 2016. Data were log_10_-transformed prior to statistical analysis to improve fit to the underlying assumptions of the Pearson’s correlation test.

**Supplementary Figure 4. Average trajectory of *Z. tritici* infection over the wheat growing season (1^st^ October – 31^st^ July) for models A1 and A2.** Successful infection refers to spore cohorts that germinate and grow hyphally along the leaf surface, leading to stomatal penetration. Infection is calculated for each hour of the growing season, as the mean of all pixels, for all growing seasons in the climate dataset (Winter 1990/Summer 1991 to Winter 2015/Summer 2016). Green and blue lines represent (sexual) ascospores and (asexual) pycnidiospores, respectively. (a) model A1. (b) model A2.

**Supplementary Figure 5. Fitting of Weibull parameters.** (a) Predicted germination (⍺ = 58.5 and γ = 1.3), (c) growth (⍺ = 189 and γ = 2.2) and (e) sporulation (⍺ = 822 and γ = 4.5) trajectories over time, at optimum temperature. In each case Weibull parameters were calculated by iterative fitting to the observed data, such that residual sums of squares (RSS) were minimised. (a) Germination and (c) growth data were obtained from Fones *et al.* (2017) [7], who assessed both spore germination and hyphal penetration of stomata on the wheat leaf surface. For germination, the data were rescaled between 0 and 1, where the greatest percentage germination at day 5 was set to 1. For growth, the first 48 hours of stomatal penetration data was excluded, and all remaining data shifted down by 48 hours; this was to account for germination taking place. We set the mean penetration at day 8 (day 10, raw data) to reflect 66% total penetration of germinated individuals. The data were then rescaled between 0 and 1. Such assumptions gave 90% penetration at 11.5 days, and 99% penetration at 15.8 days under optimal conditions. (e) Disease dynamics data were obtained from Bernard *et al*. (2013) [30] under optimal temperature class (16.6 – 18.7 ^o^C). Data were shifted by 94 hours to ensure that approximately 37% completion occurs at LP_min_ (28.8 days, 691 hours), under optimal conditions (18.4 ^o^C), thereby ensuring consistency to Equation 1. 2D contour plots showing goodness-of-fit measurements for (b) germination (RSS = 0.664), (d) growth (RSS = 0.157) and (f) sporulation. (RSS = 0.0136). Black dots refer to best fit parameter combinations.

**Supplementary Figure 6. Variation in ascospore cohort size available to land on the wheat leaf surface during a wheat growing season.** In both models A and B, we calculate relative hourly ascospore cohort size using an ascospore influx rate equation, see Kitchen *et al.* (2016) [35]. In our model, *λ* = 0.00159, approximating ascospore delivery at the temporal scale of models A and B. Vertical blue, green and red lines approximate December 1^st^, March 1^st^, and June 1^st^, respectively.

**Supplementary Figure 7. Example germination trajectory of a single spore cohort, in a single pixel, during a specific wheat growing season for model A.** (a) A spore cohort lands on the leaf surface at *t* = 441. Over time, the proportion of spores that germinate (green line) and die (red line) increases, and the proportion of spores remaining to transition (grey line) decreases. When *t* ≥ 441, the proportion of germinated, dead and remaining spores (blue line) sum to 0.4495 (total cohort size at *t* = 441). When *t* < 441, spores have not yet landed on the leaf, and so these proportions sum to 0. (b) Canopy temperature (^o^C) and (c) canopy wetness status (binary) during the time period.

**Supplementary Figure 8. Sites with available observed STB disease utilized in Figure 5.** (a) 80 locations provided observed STB disease data (% leaf area) for at least one wheat growing season (Winter 2001/Summer 2002 – Winter 2015/Summer 2016). Wheat plants were untreated with fungicide at all locations. Data were downloaded from the Agriculture and Horticulture Development Board (https://cereals.ahdb.org.uk). x and y values refer to longitude and latitude, respectively. (b) Observed STB disease for each of 80 locations. (c) Observed STB disease for each of 22 pixels.

**Supplementary Figure 9. Pearson’s correlation of per-pixel mean and standard deviation of observed STB disease (%).** (a) r = 0.621, p < 0.01; n = 21. (b) Mean and (c) standard deviation of observed STB infection (%) by location. x and y values of (b) and (c) refer to longitude and latitude, respectively. Mean observed STB disease was log_10_-transformed prior to statistical analysis to improve fit to the underlying assumptions of the Pearson’s correlation test.

**Supplementary Figure 10. Summary climate of UK wheat growing region (**1^st^ Oct 1990/31^st^ July 1991 – 1^st^ Oct 2015/31^st^ July 2016**).** (a) Mean hourly temperature (^o^C). (b) Mean temperature per pixel (^o^C). (c) Hourly fraction wet pixels. (d) Mean wetness per pixel (g m^-2^). (e) Mean hourly precipitation + 1 (mm). Y axis shows a log_10_ scale. (f) Mean hourly precipitation per pixel (mm). x and y values of (b), (d) and (f) refer to longitude and latitude, respectively. Solid lines in (a) and (c) represent annual means. Data from Kobayashi *et al.* (2015) [29].

**Supplementary Figure 11. Linear regression analysis of *in vitro* growth rate of *Z. tritici* over 15 days at varying temperatures.** Cytoplasmic fluorescence of ectopic-GFP expressing cells in 12 μm Z stack images was used as a proxy for biomass. Growth rate was estimated as the slope of the linear regression between square root(cytoplasmic fluorescence) and day. Graph labels refer to culture temperature (^o^C). Colour of data points and estimated linear regression refer to biological replicate.
